# Development and validation of an instrument to monitor obstetric care provided in maternity hospitals

**DOI:** 10.1590/0034-7167-2025-0151

**Published:** 2026-02-23

**Authors:** Fábio André Miranda de Oliveira, Clara Fróes de Oliveira Sanfelice, Tatiane Herreira Trigueiro, Maria Cristina Gabrielloni, Ariane Polidoro Dini

**Affiliations:** 1 Universidade Estadual de Campinas. Campinas, São Paulo, Brazil.; 2 Universidade Estadual do Paraná. Curitiba, Paraná, Brazil.; 3 Universidade Federal de São Paulo. São Paulo, São Paulo, Brazil.

**Keywords:** Delivery, Obstetric, Validation Study, Quality Improvement, Patient Safety, Infant, Newborn

## Abstract

**Objectives:**

to develop and validate an instrument to monitor obstetric care provided in maternity hospitals.

**Methods:**

this methodological study was conducted in two stages: (1) development of the instrument based on a narrative review and (2) content validation through evaluation of the instrument by nine experts.

**Results:**

three documents were developed: the first, a checklist with 61 monitoring items; the second, a document specifying the operational definition of each item; and the third, guidance for calculating the indicator generated by each item. The content validity index was greater than 0.78 after two rounds of data collection.

**Conclusions:**

the monitoring instrument provided evidence of content validity from experts across all assessed domains and can therefore be considered reliable for monitoring obstetric care.

## INTRODUCTION

Approximately 142 million births occur worldwide each year, most among women without risk factors for complications during childbirth. Nevertheless, childbirth remains a critical period for the survival of the mother–newborn dyad, with increased risk of morbidity and mortality if complications arise^(1)^.

Globally, two-thirds of neonatal deaths occur within the first three days of life. Most are related to complications of prematurity, intrapartum asphyxia, and neonatal infections. Providing high-quality routine care during labor, childbirth, and the immediate postpartum period is, therefore, essential to prevent newborn deaths. Thus, even in settings where skilled health professionals attend most births, it is still necessary to ensure quality practices^(2)^.

Regarding maternal mortality, multiple factors are involved, such as socioeconomic status and limited access to women’s health services, the skill and knowledge of professionals, the quality of obstetric care, and instances of disrespect and abuse of women^(3)^.

The World Health Organization (WHO) emphasizes that, to “save lives,” evidence-based practices in care for women and newborns must be properly implemented to ensure quality and safety throughout pregnancy, childbirth, and the postpartum period^(4)^.

This recommendation aligns with *Sustainable Development Goal (SDG) 3: Ensure healthy lives and promote well-being for all at all ages*, which is linked to strategies for women’s and children’s health through 2030. The global maternal mortality ratio is expected to fall to fewer than 70 deaths per 100,000 live births by 2030^(1)^.

Global agendas are increasingly focused on ensuring that women and newborns not only survive childbirth but also thrive in health and well-being. Positive experiences of women during perinatal care and childbirth strengthen the foundation for healthier motherhood^(1,4)^.

In 2018, the World Health Organization (WHO) issued the recommendation *Intrapartum care for a positive childbirth experience*, which recognizes a positive childbirth experience as a significant outcome for all women. A *positive childbirth experience* is defined as one that meets or exceeds a woman’s personal beliefs and expectations. It is associated with a healthy newborn delivered in a safe environment, with continuous practical and emotional support from a companion of her choice, along with care provided by a technically competent team^(1)^.

Continuous evaluations are essential to improving service quality and ensuring that recommended practices are effectively implemented. Therefore, health evaluation requires a reference standard to allow the appraisal of an intervention or social practice. When such standards are based on norms or guidelines derived from public policies, this is referred to as a *normative evaluation*. Evaluation models for this type of service should aim to comply with current public policies^(5,6)^.

It is recognized that services often lack instruments to compile and interpret obstetric indicators, and even more so concrete strategies to monitor such data. This study, therefore, seeks to address this gap by providing maternity hospital managers with a tool composed of maternal and neonatal health indicators that enables appropriate monitoring of obstetric care.

## OBJECTIVES

To develop and validate the content of an instrument to monitor obstetric care provided in maternity hospitals.

## METHODS

### Ethical aspects

The study complied with national and international ethical standards for research involving human participants. It was approved by the Research Ethics Committee at the University of Campinas (Unicamp). The experts invited to participate were informed about the study objectives and the nature of data collection. Those who agreed provided electronic consent by signing the Informed Consent Form (ICF). 

### Study design, setting, and period

This was a methodological study. The steps for developing and validating the instrument were observed: establishing the conceptual framework; defining the objectives and target population; constructing the items and response scale; selecting and organizing the items; structuring the instrument; and conducting content validation^(7,8)^.

A narrative review of the literature was conducted to identify key items for monitoring in obstetric practice. These materials provided the foundation for drafting a proposed instrument, which a group of experts subsequently evaluated. The study procedures were carried out from May 2022 to December 2023. Contact with the experts occurred virtually, and it was not possible to conduct a pretest of the instrument.

The preparation of this article was guided by the document *Reporting the results of studies of instrument and scale development and testing* to ensure more accurate reporting of the research findings^(9)^.

### Population or sample; inclusion and exclusion criteria

Ten experts in women’s health were invited to participate in this study. The inclusion criteria were as follows: researchers in the field of women’s health with a doctoral degree, whether or not affiliated with a higher education institution; health professionals (physicians or nurses) specialized in obstetrics who provide care for low-risk births in maternity hospitals; managers (physicians or nurses) of obstetric services for low-risk births who are specialists in obstetrics; and experts in research methodology, particularly in instrument validation.

Professionals were invited if their curricula available online met the established criteria, or if they were recommended through professional contacts. Refusal to participate was considered the exclusion criterion. Of the ten experts invited, nine agreed to participate.

### Study protocol

The narrative review resulted in the development of the theoretical framework used to construct the instrument, named the *Checklist do Cuidado Obstétrico e Neonatal* (Obstetric and Neonatal Care Checklist). This process allowed the identification of key items to be monitored in the evaluation of obstetric practice. Twenty-three institutional documents, protocols, guidelines, and other materials related to quality parameters in childbirth and neonatal care were analyzed. Evaluation documents focused on the client’s perspective were excluded, based on the understanding that these represent different viewpoints and that women’s perceptions are already well established in the literature.

Sixty-one items were identified for inclusion in the proposed Instrument for Monitoring Obstetric Practice. In addition to the existing indicators, five items were added to record information relevant to identifying the woman whose care would be assessed.

The items were organized into eight categories: two addressed identification and information related to Patient Safety in Maternity Hospitals, and the remaining six referred to the continuum of care from admission and postpartum care to newborn care.

The response scale included the options “Y” for yes, “N” for no, and “NA” for not applicable. Specific spaces were provided for items requiring descriptive responses, such as age, parity, or type of laceration.

Since indicators could be derived from the instrument, two complementary documents were developed to strengthen its use (Document 1). One defined the concepts and operational definitions of each item in the instrument (Document 2), and the other provided the basis for calculating indicators generated from the monitored items (Document 3). Document 3 was prepared with the support of a statistician to ensure that the numerator and denominator were compatible with the item being calculated.

Because this instrument was developed for the Brazilian context, the target population was defined as women receiving care during the childbirth process in a given institution.

The *Obstetric and Neonatal Care Checklist*, consisting of 61 items related to childbirth and neonatal care, was sent to the experts for evaluation. Among the eight categories in the instrument, only the identification category was not submitted for expert assessment, as it is mandatory in this type of instrument. Two additional documents were also sent for review. Thus, the three documents were shared electronically via Google Forms, along with a questionnaire to characterize the experts. The experts were asked to assess the items according to three criteria: coverage, comprehensibility, and relevance. Relevance referred to whether the items were pertinent to the construct of interest, considering the target population and context of use. Comprehensibility assessed whether the items were described clearly. Coverage verified whether each category was adequately represented by the set of items and whether all relevant dimensions were included. Experts were not asked to evaluate coverage in Document 3, as it served only as guidance for calculating indicators generated from the monitored items.

To complete this stage, the evaluation form included a Likert-type scale for each category and item, ranging from 1 to 4:(1)not relevant at all; (2)not relevant; (3)relevant; and (4) highly relevant. The simple ordinal structure of the scale allowed experts to express agreement or disagreement with each proposed item^(10)^.

### Analysis of results and statistics

The results were analyzed using simple descriptive statistics. All responses from the content validity evaluation were exported from the response platform and entered into a spreadsheet to calculate the Content Validity Index (CVI), using the formula: CVI = ∑ number of responses “3” or “4” / total number of responses ^(11)^. A CVI greater than 0.78 was considered acceptable. Thus, the agreement rate for each individual item had to exceed 0.78 (CVI > 0.78)^(11)^. Microsoft Excel was used to calculate the CVI by item and by criterion.

## RESULTS

Following the literature review, three documents were developed: the *Obstetric and Neonatal Care Checklist* (Document 1), *Operational definitions for the checklist* (Document 2), and the *Calculation basis for the indicator of each item in the instrument* (Document 3).

The documents were organized into eight evaluation categories: *Maternal safety*, *Care provided at maternal admission*, *Care provided during the first stage of labor*, *Care provided during the second stage of labor*, *Care provided during the third stage of labor*, *Care provided to the newborn*, *Care provided during the immediate postpartum period*, and *Patient identification*.

Of the ten professionals invited to evaluate the instrument’s content, only one declined to participate. The characteristics of the participants are presented in Table 1. The response deadline was one month.

**Table 1:** Characteristics of the experts who evaluated the instrument (N = 9), Campinas, São Paulo, Brazil, 2023

Characteristics	n	%
**Years of professional experience since graduation (years)**		
7-10	2	22.22%
11-20	5	55.55%
20-41	2	22.22%
**Field of practice**		
Direct care as a nurse or physician	4	44.44%
Management of obstetric services (nursing or medicine)	2	22.22%
Teaching and research	2	22.22%
**Professional qualification**		
Graduate certificate	2	22.22%
Master’s degree	3	33.33%
Doctoral degree	4	44.44%

Initially, the checklist consisted of 61 items related to childbirth and neonatal care, and it was subsequently expanded to 62 with the inclusion of a maternal near miss item suggested by the experts. Its organization into eight evaluation categories was maintained. Since the associated documents are based on the instrument, a maternal near miss item was also included in each of the three documents to maintain standardization and ensure that the instrument met its objective.

The data from the experts’ content validity evaluation are presented in Chart 1.

**Chart 1:** Items reformulated and included after expert review (N = 9), Campinas, São Paulo, Brazil, 2023

Item number	Original item	Modified/included item
	Item added	*Houve near miss materno em algum momento do processo assistencial?*
29	*Foram incentivadas a adoção de uma postura vertical e a mobilidade corporal durante o trabalho de parto?*	*Foram incentivadas a adoção de posturas verticais e a mobilidade corporal durante o trabalho de parto?*
33	*Foi adotada uma postura vertical de escolha da gestante para o parto?*	*Foi adotada uma posição/postura vertical de escolha da gestante para o parto?*
35	*O parto foi atendido por enfermeira(o) obstétrica(o)?*	*O parto foi atendido por enfermeira(o) obstétrica(o) ou obstetriz?*
46	*Se houve laceração perineal, foi suturada com uso de analgesia (local ou sistêmica)?*	*Se houve laceração perineal, com necessidade de sutura, teve uso de analgesia (local ou sistêmica)?*
49	*Foi realizada amamentação do recém-nascido em peito materno na primeira hora de vida?*	*Foi realizada amamentação do recém-nascido em seio materno na primeira hora de vida?*
57	*Foi realizada ventilação por pressão positiva nos primeiros 10 minutos após o nascimento?*	*Foi realizada ventilação por pressão positiva nos primeiros 10 minutos de vida?*

Due to the large number of items in each document, the mean CVI by criterion and by document was highlighted. This approach was considered sufficient to represent the CVI for each item. In Document 1, the mean CVI was 0.954 for comprehensibility, 0.967 for relevance, and 0.958 for coverage. In Document 2, the mean CVI was 0.969 for comprehensibility, 0.980 for relevance, and 0.952 for coverage. In Document 3, the mean CVI was 0.858 for comprehensibility and 0.890 for relevance. Coverage for all categories was equal to or greater than 0.89. These results are shown in Table 2.

**Table 2:** Distribution of the documents according to the Content Validity Index (CVI) by criterion evaluated by the experts, Curitiba, Paraná, Brazil, 2023

	Comp*	Rel*	Cov*	*S-CVI
Document 1	0.954	0.967	0.958	0.96
Maternal safety			0.89	
Care provided at maternal admission			1.0	
Care provided during the first stage of labor			0.89	
Care provided during the second stage of labor			1.0	
Care provided during the third stage of labor			1.0	
Care provided to the newborn			0.89	
Care provided during the immediate postpartum period			1.0	
Document 2	0.969	0.980	0.952	0.97
Maternal safety			1.0	
Care provided at maternal admission			1.0	
Care provided during the first stage of labor			0.89	
Care provided during the second stage of labor			1.0	
Care provided during the third stage of labor			1.0	
Care provided to the newborn			0.89	
Care provided during the immediate postpartum period			0.89	
Document 3	0.858	0.890	**	0.87

* *Comp – comprehensibility; Rel – relevance; Cov – coverage; S-CVI – scale-level Content Validity Index;*

** *Coverage was not assessed for this document*.

## DISCUSSION

Monitoring childbirth and neonatal care through indicators is an essential strategy for identifying nonconformities and providing appropriate and timely interventions to improve care^(12)^.

There is a scarcity of updated and validated audit instruments for obstetric services, such as the one proposed in this study. Consequently, it is challenging to find chart review checklists suited to the reality of obstetric practices in Brazil^(13)^. Many evaluations arise from external demands, such as academia or regulatory agencies. In addition, international documents that could serve as a basis for evaluation are often summarized and do not adequately reflect the reality of the care assessed^(13,14)^.

Serving as the basis for the other documents, Document 1 linked each category to a specific need for assessing care quality. The category *Maternal safety* is justified by the fact that it is a relevant topic in care quality today. Since 2013, Resolution RDC No. 36 has established^(15)^ actions for patient safety in health services. These include implementing protocols that promote patient safety, monitoring care indicators, and developing and implementing strategies aimed at improving the quality of health services. In 2014, Anvisa issued a document focused on safety and quality in maternity services, describing “sentinel events or incidents that should trigger actions for analysis and evaluation”^(16)^. Accordingly, the formulated items are directly related to this need and are supported by the proposed indicators, which are essential for the health of the mother–newborn dyad.

The categories *Care provided at maternal admission*, *Care provided during the first stage of labor*, *Care provided during the second stage of labor*, *Care provided to the newborn*, and *Care provided during the third stage of labor* describe maternal physiology in the childbirth process linearly and consistently. Their items were designed based on national and international protocols, as well as current recommendations for best practices in childbirth and neonatal care(1,17,18). To incorporate more recent scientific evidence, two protocols from the Brazilian Society of Pediatrics were included in the category *Care provided to the newborn* ^(19,20)^.

The study objective was achieved through the choice of the validation method and the steps adopted for the development of the instrument^(7)^.

The use of a group of experts to validate the instrument was appropriate, as their consensus evaluation contributed to the development of a more reliable instrument based on their expertise. The group was initially composed of ten specialists in the field (according to pre-established criteria), but only one did not return the evaluation. This loss did not compromise the continuity of the study, since nine experts constitute a sample size considered adequate in the literature^(11,21,22)^.

The content validation criteria adopted for the instrument and its items were comprehensibility, relevance, and coverage, which are commonly reported in the literature^(21,23-25)^. The positive evaluation by the experts demonstrates that the items of the instrument are consistent with clinical practice^(26)^.

Comprehensibility makes it possible to assess whether the items are written clearly and intelligibly, ensuring the instrument’s adequacy for the observed event^(27)^. Among the various validation criteria, relevance stands out and is frequently cited as one of the main indicators of an item’s content validity^(26)^. Coverage, in turn, determines whether the categories or domains are properly represented by the items they comprise^(11)^.

Because the group of experts exceeded six individuals, a cutoff point of 0.78 was recommended for the CVI. This was the threshold used to accept an item, whether reformulated or not. In this study, none of the items in the three documents presented a CVI below 0.78. In other words, at least seven of the nine experts rated each item as “3” or “4” in the item-level analysis. Thus, it was not necessary to exclude any item due to inconsistent CVI. Several studies have reported using this cutoff as a quality parameter for validating measurement instruments^(22,24,25)^.

There is no consensus on how to evaluate the instrument as a whole. One approach described in the literature is to calculate the mean of the CVI values of all items, summing the CVI for each and dividing the result by the total number of items considered^(28)^. The literature partly diverges on the minimum acceptable validity value for the instrument as a whole, with reports ranging from 0.80 to 0.90 for the scale-level CVI^(11,28)^.

In the calculation of the CVI by item, by criterion, and overall, the results of the experts’ analysis were high and consistent with the concepts in the literature that guided this study^(28)^.

In Document 3, coverage was not calculated because it was intended to guide the calculation of indicators derived from the instrument. Thus, its evaluation focused on the comprehensibility and relevance of the items. As in the other documents, no item had a CVI below 0.78, which led to the retention of all initial items.

It is worth emphasizing that all three documents are interconnected and designed so that each item complements the previous one with respect to the instrument’s objective. Although the evaluation of Document 3 ended up differing from those related to Documents 1 and 2, all items were maintained, as they individually exceeded the minimum CVI of 0.78. When analyzed by criterion, Document 3 showed a CVI of 0.86 for comprehensibility and 0.89 for relevance. The scale-level CVI for this document was 0.87. These values confirm the validity of the Document 3 and of the previous ones, since the validation of this document considered an S-CVI greater than 0.80^(11,28)^.

Although the CVI values per item were equal to or greater than 0.78, the experts’ suggestions were incorporated. These suggestions improved the clarity and readability of the items without substantially altering their definition or concept.

The prominent inclusion was an item related to the occurrence of maternal near miss. All experts mentioned the need to include an item to monitor this adverse maternal health event. The WHO defines *maternal near miss* as a “near-death” event in which a woman nearly died but survived a complication that occurred during pregnancy, childbirth, or within 42 days of termination of pregnancy^(29)^. Its highest occurrence is known to be associated with care provided during labor and childbirth^(29)^.

Because this event involves the entire care process, the experts’ request was incorporated at the end of the document, ensuring that its initial structure was not altered. It was described in Document 1 as *Houve near miss materno em algum momento do processo assistencial?* (Was there a maternal near miss at any point during the care process?); in Document 2, as *Houve near miss materno em algum momento do processo assistencial* (Was there a maternal near miss at any point during the care process); and in Document 3, as *Percentual de near miss materno* (Percentage of maternal near miss), following the implementation manual’s guidance for calculating this indicator.

The textual changes were minor but well justified. In Item 29, the term *postura vertical* (upright postures) was placed in the plural, while in Item 33, the word *posição* (position) was added in association with *posture*. Among the WHO-recommended good practices for a positive childbirth experience is the adoption of upright postures and mobility of the woman in labor during both labor and delivery. These practices should be encouraged and supported by the care team^(1)^.

The inclusion of the midwife in Item 35 was justified by her legal authority to provide childbirth and neonatal care, as per Resolution 516/2026 of the Federal Nursing Council^(30)^. Models of continuous care provided by nurse-midwives or midwives have been shown to be safe and to result in greater satisfaction among women regarding the care they receive^(31)^. It is, therefore, essential to determine whether these professionals are providing appropriate care.

Current care protocols emphasize that the professional should assess the need for perineal suturing and, depending on the degree, make the decision together with the woman. Not all lacerations necessarily require suturing^(17,18)^. Thus, the suggested modification to Item 46 was considered relevant. Regarding Items 49 and 57, only the wording was modified to improve clarity.

The research team decided not to conduct a new round with the experts, since the CVI results were adequate for all established evaluation criteria. The experts’ suggestions regarding wording were incorporated, but they did not alter the format or concept of the instrument, and the inclusion of the suggested item was unanimous.

The difficulties encountered in the study were directly related to the lack of consistency in the literature regarding the concept and definition of *validation of research instruments*.

### Study limitations

The pretest stage was not conducted, which represents a limitation of this study. The process of developing an instrument is complex and continuous. Therefore, the instrument should be further examined for construct validity, criterion validity, and reliability.

### Contributions to the field

The main contribution of this study is advancing research on the development and adaptation of a specific instrument to evaluate obstetric care provided in maternity hospitals.

This instrument appears to address aspects related to childbirth and neonatal care practices, as well as to identify events potentially significant for the health of the mother–newborn dyad. It is a user-friendly tool intended to support health professionals in improving obstetric practices and in achieving the Sustainable Development Goals.

In the absence of other instruments designed to assess the quality of low-risk obstetric practices, the use of the instrument validated in this study is recommended.

## CONCLUSIONS

This study enabled the development and dissemination of a checklist and two supporting documents with evidence of content validity for monitoring obstetric care provided in maternity hospitals.

The use of these three documents can equip institutions to assess the quality and safety of care for women and newborns, considering that maternal and neonatal health are important indicators of the quality of life of a given population. In recent decades, a significant movement by women, government agencies, and non-governmental organizations has advocated for respectful and qualified care for women and their children.

It is recommended that future studies apply the instrument, thereby enabling an evaluation of its performance in practice. 

## Data Availability

The research data are available only upon request.
