# Supplementary material for: Development and validation of an instrument to monitor obstetric care provided in maternity hospitals
Source: Rev Bras Enferm. 2026 Feb 23;79:e20250151. doi: 10.1590/0034-7167-2025-0151 (PMC12933930; doi:10.1590/0034-7167-2025-0151)
Supplement: Translation

# Translation: Construção e validação de um instrumento para monitorar a assistência obstétrica realizada em maternidades

**DOI:** 10.1590/0034-7167-2025-0151pt

**Authors:** Fábio André Miranda de Oliveira, Clara Fróes de Oliveira Sanfelice, Tatiane Herreira Trigueiro, Maria Cristina Gabrielloni, Ariane Polidoro Dini

**Affiliations:** I Universidade Estadual de Campinas, Campinas, São Paulo, Brasil, Universidade Estadual de Campinas. Campinas, São Paulo, Brasil; II Universidade Estadual do Paraná, Curitiba, Paraná, Brasil, Universidade Estadual do Paraná. Curitiba, Paraná, Brasil.; III Universidade Federal de São Paulo, São Paulo, São Paulo, Brasil, Universidade Federal de São Paulo. São Paulo, São Paulo, Brasil.

**Keywords:** Parto Humanizado, Estudo de Validação, Melhoria de Qualidade, Segurança do Paciente, Recém-Nascido

## Abstract

**Objetivos:**

construir e validar um instrumento para monitorar a assistência obstétrica realizada em maternidades.

**Métodos:**

estudo metodológico realizado em duas etapas: 1) construção do instrumento com base na revisão narrativa; e 2) validação deconteúdo mediante a avaliação do instrumento por nove juízes.

**Resultados:**

foram construídos três documentos: o primeiro, um checklist com 61 itens de monitoramento; o segundo, um documento definindo cada item; e o terceiro, uma orientação para cálculo de indicador fornecido por cada item. Obteve-se o índice de validade de conteúdo superior a 0,78 após duas rodadas de coleta de dados.

**Conclusões:**

o instrumento de monitoramento apresentou evidências de validade de conteúdo por juízes em todos os quesitos analisados; logo, mostra-se seguro para o monitoramento da assistência obstétrica.

## INTRODUÇÃO

Aproximadamente 142 milhões de nascimentos acontecem por ano no mundo, sendo a maioria proveniente de mulheres sem fatores de risco de complicações para o parto. Mesmo assim, o nascimento ainda é um momento crítico para a sobrevivência do binômio, com risco aumentado de morbilidade e mortalidade se surgirem complicações^1^.

Globalmente, dois terços das mortes neonatais ocorrem nos três primeiros dias de vida. A maioria delas estão relacionadas a complicações de prematuridade, asfixia intraparto e infecções neonatais. Entende-se que realizar cuidados de rotina de alta qualidade durante o trabalho de parto, parto e pós-parto imediato é imprescindível para a prevenção de mortes de recém-nascidos. Assim, mesmo em locais onde a maioria dos nascimentos são assistidos por profissional de nível superior, ainda é preciso garantir práticas de qualidade^2^.

No tocante à mortalidade materna, estão associados múltiplos fatores, como nível socioeconômico e acesso limitado aos serviços de saúde da mulher, nível de habilidade e conhecimento dos profissionais, qualidade da assistência ao parto e aspectos de desrespeito e violência às mulheres^3^.

A Organização Mundial de Saúde (OMS) destaca que, para “salvar vidas”, faz-se necessário implementar adequadamente práticas baseadas em evidências na assistência à mulher e ao recém-nascido, de modo que garantam a qualidade e segurança dos cuidados durante toda a gestação, parto e puerpério^4^.

Tal afirmação alinha-se com o “Objetivo de Desenvolvimento Sustentável (ODS) 3: Assegurar uma vida saudável e promover o bem-estar para todos, em todas as idades”, associado a estratégias para a saúde das mulheres e crianças até 2030. Pretende-se a redução da taxa global de mortalidade materna para números inferiores a 70 mortes por 100.000 nascidos vivos até 2030^1^.

As agendas globais estão ampliando seu foco para garantir que mulheres e recém-nascidos não somente sobrevivam ao processo de nascimento, mas também prosperem em sua saúde e qualidade de vida. Logo, experiências positivas das mulheres durante os cuidados perinatais e parto fortalecem a base de uma maternidade mais saudável^1,4^.

Em 2018, a recomendação *Intrapartum care for a positive childbirth experience*, da OMS, reconhece a importância de uma experiência positiva no parto como um resultado significativo para todas as mulheres. Define como “experiência positiva de parto” aquela que cumpre ou supera crenças e expectativas pessoais da parturiente. Associa-se a esse evento o nascimento saudável em ambiente seguro com contínuo apoio prático e emocional de um companheiro de sua escolha, além de uma equipe tecnicamente competente^1^.

Avaliações contínuas são indispensáveis para a melhoria da qualidade dos serviços, a fim de garantir o cumprimento das práticas propostas. Desse modo, a avaliação em saúde necessita de parâmetro de referência para que se possa emitir juízo de valor acerca de alguma intervenção ou prática social. Quando tais parâmetros estão baseados em normas ou diretrizes oriundos de políticas públicas, trata-se de uma “avaliação normativa”. Modelos de avaliação para esse tipo de serviço devem ter como meta atender às políticas públicas vigentes^5,6^.

Entende-se que os serviços, muitas vezes, não dispõem de instrumentos para compilar e interpretar indicadores obstétricos e muito menos de estratégias concretas para monitorar esses dados. Assim sendo, esta pesquisa visa sanar uma lacuna do processo de trabalho oferecendo aos gestores de maternidades uma ferramenta composta por indicadores em saúde materna e neonatal que permita um monitoramento adequado da assistência.

## OBJETIVOS

Construir e validar o conteúdo de um instrumento para monitorar a assistência obstétrica realizada em maternidades.

## MÉTODOS

### Aspectos éticos

O estudo atendeu às normas nacionais e internacionais de ética em pesquisa envolvendo seres humanos. A pesquisa obteve aprovação do Comitê de Ética em Pesquisa da Universidade Estadual de Campinas (Unicamp). Os especialistas participantes foram esclarecidos quanto ao objetivo e natureza da coleta de dados da pesquisa. Aqueles que concordaram assinaram eletronicamente o Termo de Consentimento Livre e Esclarecido.

### Desenho, local do estudo e período

Trata-se de um estudo metodológico. Respeitaram-se as etapas para construção e validação de instrumento: estabelecimento da estrutura conceitual; definição dos objetivos e população; construção dos itens e da escala de resposta; seleção e organização dos itens; estruturação do instrumento; e validação de conteúdo^7,8^.

Para a construção do instrumento, foi realizada revisão narrativa da literatura, que resultou na identificação de itens importantes a serem monitorados na prática obstétrica. Os materiais encontrados serviram de base para a confecção de uma proposta de instrumento, que posteriormente foi avaliada pelo grupo de juízes especialistas. As etapas transcorreram de maio de 2022 a dezembro de 2023. O contato com os juízes ocorreu virtualmente. Não foi possível a realização de pré-teste do instrumento.

A construção deste artigo amparou-se no documento *Reporting the results of studies of instrument and scale development and testing*, para relatar melhor os resultados de pesquisa^9^.

### População ou amostra; critérios de inclusão e exclusão

Para este estudo, foram convidados dez juízes especialistas na área de saúde da mulher. Foram estabelecidos como critérios de inclusão: pesquisadores na área de saúde da mulher, com título de doutorado, pertencentes ou não a instituição de ensino superior; profissionais de nível superior (médico ou enfermeiro) com especialidade em obstetrícia e que assistem ao parto de risco habitual em maternidades; gestores de nível superior (médico ou enfermeiro) de serviços de obstetrícia de risco habitual que sejam especialistas em obstetrícia; e especialistas em metodologia de pesquisa, especialmente na temática da validação de instrumentos.

Foram convidados profissionais cujos currículos disponíveis na internet atendiam aos critérios estabelecidos ou que foram indicados por contatos profissionais. Foi considerado critério de exclusão a recusa a participar da pesquisa. Dos dez juízes convidados, nove aceitaram participar.

### Protocolo do estudo

A revisão de narrativa resultou na construção do referencial teórico a ser utilizado na construção do instrumento, denominado “Checklist do Cuidado Obstétrico e Neonatal”. Assim, itens importantes a serem monitorados no processo de avaliação da prática obstétrica foram identificados. Foram analisados 23 documentos institucionais, protocolos, diretrizes e demais materiais relacionados com parâmetros da qualidade na assistência ao parto e nascimento. Foram excluídos documentos avaliativos centrados na perspectiva da cliente. Essa afirmação se apoia na compreensão de que são olhares distintos e de que a percepção das mulheres já está bem definida na literatura.

Foram identificados 61 itens a serem incluídos na proposta do instrumento de Monitorização da Prática Obstétrica. Aos indicadores existentes, adicionaram-se cinco informações pertinentes à identificação da parturiente a ter a assistência avaliada.

Os itens foram organizados em oito categorias: duas destacam a identificação e informações referentes à Segurança do Paciente em Maternidades. As demais seis categorias referem-se ao processo que vai da admissão da parturiente e cuidados puerperais até a assistência realizada ao recém-nascido.

A escala de respostas foi organizada com as opções “S” para sim, “N” para não e “NA” para não se aplica. Cabe destacar que foram deixados espaços específicos para alguns itens que requerem respostas descritivas específicas, como idade, paridade ou tipo de laceração.

Entendendo que indicadores poderiam ser formados com autilização do instrumento, idealizaram-se dois documentos acessórios para deveras dispensar apoios teóricos. O um, deno-minado “definições conceituais e contexto gententico e conceito de cada item do instrumento (Documento 2). O outro fornece a base de cálculo para tabulação dos indicadores oriundos dos itens a serem monitorados (Documento 3). Esse Documento 3 foi confeccionado com auxílio de um estatístico, para que o numerador e o denominador fossem compatíveis com o item a ser calculado.

Por se tratar de uma realidade brasileira, determinou-se como população-alvo do instrumento mulheres atendidas que vivenciam o atendimento no processo de parto e nascimento em determinada instituição.

Foi enviado para avaliação dos juízes o “Checklist do Cuidado Obstétrico e Neonatal” com 61 itens relacionados ao processo de assistência ao parto e nascimento. As oito categorias presentes no instrumento, apenas a categoria de identificação não foi colocada para avaliação, por ser de natureza descritiva e obrigatória nesse tipo de instrumento. Outros documentos foram encaminhados nos seguintes três documentos adicionais enviados de forma eletrônica, via Google Forms, juntamente com um questionário para coleta de características. Solicitou-se que eles avaliassem os critérios de abrangência, compreensibilidade e relevância dos itens. A relevância refere-se ao fato de os itens serem relevantes para o construto de interesse, de acordo com a população e contexto de uso. A compreensibilidade avalia se os itens estão descritos de forma compreensível. Já a abrangência diz respeito a verificar se cada categoria é representada pelo conjunto de itens e se todas as dimensões de cada item são incluídas, solicitou-se que os juízes avaliassem o critério de abrangência no Documento 3, por ele ser uma orientação para cálculo de indicadores oriundos dos itens monitorados.

Para cumprir essa etapa, o formulário de avaliação dos juízes continua com a escala de 1 a 4 do tipo Likert para cada categoria e item avaliados, variando de 1 a 4 pontos: (1) definitivamente não relevante; (2) não relevante; (3) relevante; e (4) definitivamente relevante. Os juízes puderam registrar sua concordância ou discordancia ao item proposto devido à sua organização simples e de caráter ordinal^10^.

### Análise dos resultados e estatística

Os resultados encontrados foram analisados com uso de estatística descritiva simples. Todas as respostas obtidas na avaliação da validade de conteúdo foram extraídas da plataforma de respostas e tabuladas em planilha eletrônica para calcular o IVC, de acordo com a fórmula: IVC =Σ Número de respostas “3” ou “4” / Número total de respostas^(11)^. Foi considerado adequado um IVC maior que 0,78. Dessa forma, a taxa de concordância deveria ser superior a 0,7840 (IVC > 0,78) na avaliação de cada item individualmente^(11)^. No tratamento dos dados, foi utilizado o Microsoft Excel para calcular os índices de validade de conteúdo (IVC) por item e por critério.

## RESULTADOS

Após a revisão da literatura, foram elaborados três documentos: “Checklist do Cuidado Obstétrico e Neonatal” (Documento 1), “Definições operacionais para o checklist” (Documento 2) e “Base de cálculo para o indicador de cada item do instrumento (Documento 3).

Os documentos foram organizados em oito categorias de avaliação: “Segurança da parturiente”; “Assistência realizada na admissão da parturiente”; “Assistência realizada no primeiro estágio do parto”; “Assistência realizada no segundo estágio do parto”; “Assistência realizada no terceiro estágio do parto”; “Assistência realizada ao recém-nascido” e “Assistência realizada no pós-parto imediato” e “Identificação da paciente”.

Dos profissionais convidados a julgar o conteúdo do instrumento, apenas um não quis participar. A caracterização dos participantes que apresentada na Tabela 1. O prazo estabelecido foi de um mês.

**Tabela 1:** Caracterização dos especialistas participantes da avaliação do instrumento (N = 9), Campinas, São Paulo, Brasil, 2023

Caracterização	n	%
**Tempo de atuação desde a formatura (anos)**		
7-10	2	22,22%
11-20	5	55,55%
20-41	2	22,22%
**Área de atuação**		
Assistência direta como enfermeiro ou médico	4	44,44%
Gerência de serviço de obstetrícia (enfermagem ou medicina)	2	22,22%
Ensino e pesquisa	2	22,22%
**Qualificação profissional**		
Especialização	2	22,22%
Mestrado	3	33,33%
Doutorado	4	44,44%

A principio, o checklist foi idealizado com 61 itens relacionados ao processo de assistência ao parto e nascimento e foi ampliado para 67, com a inclusão do item referente à ocorrência de *near miss* materno, sugerindo pelos especialistas. Mesmo assim, foi mantida a organização em oito categorias. Os três documentos associados são baseados no instrumento, um item de referência na organização de *near miss* materno foi adicionado aos documentos, a fim de manter a padronização das informações e garantir que o instrumento cumpra com o seu objetivo.

Os dados obtidos das respostas dos especialistas na avaliação da validade de conteúdo estão apresentados no Quadro 1.

**Quadro 1:** Itens reformulados e incluídos após análise dos especialistas (N = 9), Campinas, São Paulo, Brasil, 2023

Nº do item	Item original	Item modificado/incluído
	Item inserido	Houve *near miss* materno em algum momento do processo assistencial?
29	Foram incentivadas a adoção de uma postura vertical e a mobilidade corporal durante o trabalho de parto?	Foram incentivadas a adoção de posturas verticais e a mobilidade corporal durante o trabalho de parto?
33	Foi adotada uma postura vertical de escolha da gestante para o parto?	Foi adotada uma posição/postura vertical de escolha da gestante para o parto?
35	O parto foi atendido por enfermeira(o) obstétrica(o)?	O parto foi atendido por enfermeira(o) obstétrica(o) ou obstetriz?
46	Se houve laceração perineal, foi suturada com uso de analgesia (local ou sistêmica)?	Se houve laceração perineal, com necessidade de sutura, teve uso de analgesia (local ou sistêmica)?
49	Foi realizada amamentação do recém-nascido em peito materno na primeira hora de vida?	Foi realizada amamentação do recém-nascido em seio materno na primeira hora de vida?
57	Foi realizada ventilação por pressão positiva nos primeiros 10 minutos após o nascimento?	Foi realizada ventilação por pressão positiva nos primeiros 10 minutos de vida?

Devido ao volume elevado de itens de cada documento, optouse por destacar o IVC médio por critério e por documento. Entende-se que essa forma permite representar o IVC de cada item. A avaliação do Documento 1 destacou um IVC médio de 0,954 para a compreensibilidade, 0,967 para a relevância e 0,958 para a abrangência. No Documento 2, compreensibilidade, relevância e abrangência tiveram seu IVC médio de 0,969; 0,980; e 0,952, respectivamente. No Documento 3, o IVC médio entre as respostas foi de 0,858 para a compreensibilidade e 0,890 para a relevância. A abrangência para todas as categorias foi igual ou superior a 0,89. Essas informações estão em destaque na Tabela 2.

**Tabela 2:** Distribuição dos documentos de acordo com o Índice de Validade de Conteúdo (IVC) por critério utilizado pelos juízes, Curitiba, Paraná, Brasil, 2023

	Comp*	Rel*	Abr*	IVC Geral
Documento 1	0,954	0,967	0,958	0,96
Segurança da parturiente			0,89	
Assistência realizada na admissão da parturiente			1,0	
Assistência realizada no primeiro estágio do parto			0,89	
Assistência realizada no segundo estágio do parto			1,0	
Assistência realizada no terceiro estágio do parto			1,0	
Assistência realizada ao recém-nascido			0,89	
Assistência realizada no pós-parto imediato			1,0	
Documento 2	0,969	0,980	0,952	0,97
Segurança da parturiente			1,0	
Assistência realizada na admissão da parturiente			1,0	
Assistência realizada no primeiro estágio do parto			0,89	
Assistência realizada no segundo estágio do parto			1,0	
Assistência realizada no terceiro estágio do parto			1,0	
Assistência realizada ao recém-nascido			0,89	
Assistência realizada no pós-parto imediato			0,89	
Documento 3	0,858	0,890	**	0,87

* *Comp – compreensibilidade; Rel – relevância; Abr – abrangência;*

** *Não se solicitou avaliação de abrangência para este documento*.

## DISCUSSÃO

O monitoramento da assistência realizada ao parto e nascimento com o uso de indicadores é uma estratégia essencial para identificar não conformidades, além de proporcionar intervenções adequadas e oportunas na melhoria dessa assistência^(12)^.

Ressalta-se a escassez de instrumentos atualizados e validados de avaliação da qualidade em obstetrícia, nos moldes do presente estudo. Dessa forma, é difícil encontrar checklists de prontuários adequados e instituídos nas maternidades obstétricas do Brasil. Muitas avaliações surgem de demandas extrainstitucionais, como da academia e do Ministério da Saúde. Além disso, os documentos internacionais que poderiam servir como base para uma avaliação são resumidos e não conseguem refletir a realidade da assistência brasileira avaliada^(13,14)^.

Tomando como base para todos os documentos, o Documento 1 teve como proposta apresentar um instrumento elaborado na proposta do instrumento de Monitorização da Prática Obstétrica. Aos indicadores oriundos, adicionaram-se cinco informações pertinentes à identificação da paciente e da assistência obstétrica prestada. Os itens foram organizados em oito categorias: oito descrevem a identificação da paciente e 53 afirmativas referentes às ações em Maternidades. As demais seis categorias referem-se ao processo de assistência à saúde desde o primeiro estágio do parto até a assistência atualmente. Desde 2013, a RDC n° 36 institui^(15)^ ações para os serviços de atenção materna e neonatal. Dentre elas, destacam-se a implantação de protocolos que promovam a segurança de pacientes, a base de indicadores de qualidade, de processos, além do desenvolvimento e implementação de estratégias direcionadas à qualidade dos serviços de saúde. Em 2014, a Anvisa lançou um protocolo para prevenção de morbimortalidade nos serviços de maternidade, descrevendo “eventos-sentinela ou indicadores de resposta inadequada” associados ao *near miss* ou óbito^(19)^. Assim, os itens formulados relacionam-se diretamente com essa necessidade, não só nos indicadores propostos que se referem às fontes de necessidades atribuíveis na saúde, em espe-essências para a saúde do binômio.

A categoria “Assistência realizada na admissão da parturiente”, “Assistência realizada no primeiro estágio do parto”, “Assistência realizada no segundo estágio do trabalho de parto”, “Assistência realizada no terceiro estágio do trabalho de parto”, “Assistência realizada ao recém-nascido” e “Assistência realizada ao recém-nascido” e “Assistência realizada no pós-parto imediato” destaca uma forma linear e comum à fisiologia materna durante o processo de entropia atá a saída do recém-nascido, com base em protocolos nacionais e internacionais, bem como em recomendações atuais em documentação científica sobre o parto e nascimento^(1,17,18)^. Para acrescentar evidências científicas em suas questões mais atuais, pelos protocolos da sociedade Brasileira de Pediatria e Sociedade Brasileira de Obstetrícia, a um instrumento proposto para a categoria “Assistência realizada ao recém-nascido” foram utilizados^19,20^.

O alcance do objetivo proposto se deu com a escolha do método de validação e as etapas para a construção do instrumento^(7)^.

A utilização do grupo de juízes para validar o instrumento foi adequada, uma vez que sua avaliação contribui efetivamente para a construção de um instrumento mais confiável, baseada em sua expertise. Do grupo de juízes, inicialmente composto por dez especialistas na área requerida nos critérios pré-estabelecidos), apenas um não retornou a avaliação. Essa perda não compromete a representatividade das respostas obtidas e o tamanho amostral considerado adequado pela literatura^(11,21,22)^.

Os critérios de validação de conteúdo escolhidos para a validar o instrumento e seus itens foram a compreensibilidade, a relevância e a abrangência, que são muito comuns em vários trabalhos encontrados^(23,24)^. A avaliação positiva realizada pelos juízes demonstra que os itens do instrumento adequam-se à prática clínica^(26)^.

A compreensibilidade permite identificar se estão redigidos de modo claro e inteligível, possibilitando tornar o instrumento adequado ao evento observado^(27)^. Diante dos vários critérios para validação existentes, destaca-se a relevância, frequentemente usado como um dos principais medidores de validade de conteúdo de um item^(26)^. Já a abrangência determina se as categorias ou domínios estão corretamente representados pelos itens que os compõem^(11)^.

Sendo o grupo de juízes superior a seis indivíduos, é recomendado um valor maior ou igual a 0,78 no resultado do IVC. Este foi o valor de corte utilizado para aceitar um item, reformulando-o quando necessário. Nesta pesquisa, nenhum item dos três documentos apresentou um IVC inferior a 0,78. Em outras palavras, pelo menos sete dos nove juízes assinalaram respostas “3” ou “4” em todas as respostas da análise por item. Desse modo, não foi necessário retirar nenhum item devido a um IVC inconvincente. Vários trabalhos demonstraram utilizar esse valor de IVC como parâmetro de qualidade para validar instrumentos de medida^(22,24,25)^.

Não existe consenso sobre a forma de avaliar o instrumento como um todo. Umas das possibilidades descritas na literatura é a média dos valores de cada item, somando o IVC de cada item e dividindo o resultado pelo número de itens considerados na avaliação^(26)^. A literatura diverge em parte sobre o valor mínimo de validade para o instrumento como um todo, sendo encontrados valores entre 0,80 e 0,90 para o IVC geral^(11,28)^.

No cálculo do IVC por item, por critérios e geral, o resultado da análise dos juízes foi elevado, respeitando o conceitos da literatura norteadores desta pesquisa^(28)^.

Já no Documento 3, optou-se por não calcular a abrangência, por este ser um documento que norteará a base de cálculo dos indicadores remanescentes do instrumento. Assim, sua relação avaliativa se dá com a compreensibilidade e com a relevância dos itens. Similarmente aos outros documentos, nenhum item apresentou IVC inferior a 0,78, o que nos levou à manutenção de todos os itens iniciais.

Vale ressaltar que todos os três documentos são inteligados e direcionados para que cada item seja complementar ao anterior ou que sirvam aos objetivos do instrumento. A avaliação referente ao Documento 3 acabou sendo diferente daquelas relacionadas ao Documentos 1 e 2. Mesmo assim, todos os itens foram mantidos, pois individualmente superaram o IVC mínimo de 0,78. Se analisado por critério, o Documento 3 apresentou um IVC de 0,86 para a compreensibilidade e 0,89 para relevância. O IVC geral desse documento foi de 0,87. Tais valores determinam a validade desse documento e dos anteriores, pois, para a validação do Documento 2, levou-se em consideração um IVC geral superior a 0,80^(11,28)^.

Mesmo com valores de IVC por item quais ou superiores a 0,78, as sugestões feitas pelos juízes foram acatadas. Elas melhoraram a escrita e a compreensão do item, mas sem promover grandes mudanças em sua definição ou conceito.

A principal inclusão foi a do item relacionado com a ocorrência de *near miss* materno. Todos os juízes mencionaram a necessidade de incluir um item de acompanhamento da ocorrência desse evento adverso à saúde materna. “*Near miss* materno” é definido pela OMS como sendo “uma mulher que quase morreu, mas que sobreviveu a complicações graves ocorridas durante o período que vai da gestação até os 42 dias de pós-parto^(29)^. Sabe-se que sua maior ocorrência está relacionada com a assistência realizada no trabalho de parto e parto^(29)^.

Por ser um evento envolvente todo o processo assistencial, a solicitação dos juízes foi inserida ao fim do documento, permitindo não só a incorporação da informação de sua ocorrência, no Documento 1 como “Houve *near miss* materno em algum momento do processo assistencial?”; e no Documento 2, como “Houve *near miss* materno em algum momento do processo assistencial?”; e no Documento 3 como “Percentual *near miss* materno?” seguindo a orientação do manual de implementação para cálculo desse indicador.

As alterações de texto foram pontuais, mas certamente justificáveis. No item 29, o termo “postura vertical” foi colocado no plural, assim como no item 33 foi acrescentada a palavra “posição”, associada à postura. Dentre as boas práticas recomendadas pela OMS para uma experienência positiva de parto, está a adoção de posturas verticais e mobilidade da parturiente tanto no trabalho de parto como no período expulsivo. As parturientes devem ser encorajadas e apoiadas pela equipe assistencial^(1)^.

Justifica-se a inclusão da obstetriz no Item 35 em razão de seu respaldo legal para atuar na assistência ao parto e nascimento com base na Resolução 516/2026 do Conselho Federal de Enfermagem^(30)^. Destaca-se que modelos de assistência contínua por enfermeiras obstétricas ou obstetrizes apresentam segurança e maior satisfação para as mulheres no tocante à assistência recebida^(31)^. Desse modo, é imprescindível compreender se esses profissionais estão realizando a devida assistência.

Atualmente os protocolos assistenciais destacam que cabe ao profissional avaliar a necessidade de sutura perineal, levando em conta do seu grau, tomar a decisão juntamente com a mulher. Não necessariamente todas as lacerações precisarão ser sutura^(17,18)^. Desse modo, foi relevante a sugestão de alteração do item 46. Quanto aos itens 49 e 57, IVC modificada apenas a escrita para melhorar a compreensão.

A equipe de pesquisa optou por não realizar nova rodada com os juízes, pois o resultado do IVC foi adequado em todos os critérios estabelecidos de avaliação. As sugestões dos juízes relacionados a escrita foram acatadas, mas não alteraram o formato do instrumento nem o seu conceito, e a inclusão do item sugerido foi unânime.

As dificuldades encontradas na pesquisa relacionaram-se diretamente à falta de homogeneidade da literatura no que diz respeito ao conceito e definição de “validação de instrumentos de pesquisa”.

### Limitações do estudo

A etapa de pré-teste não foi realizada, sendo uma limitação desta pesquisa. O processo de desenvolvimento de um instrumento é complexo e contínuo. Dessa forma, o instrumento deverá ser analisado quanto à validade de construto ou critério e confiabilidade.

### Contribuições para a área

Como contribuição principal, podemos citar o avanço nos estudos da temática de adequação e criação de instrumento específico para avaliar a assistência obstétrica realizada em maternidades.

Acredita-se que o instrumento consegue contemplar os aspectos relativos às práticas da assistência ao parto e nascimento, além de identificar eventos possivelmente significativos para a saúde do binômio. Dessa forma, o instrumento pode auxiliar compreensão seu objetivo de apoiar profissionais de saúde na melhora das práticas obstétricas de cuidado no âmbito da Saúde Pública e dos Objetivos de Desenvolvimento Sustentável.

Diante da ausência de outros instrumentos com a finalidade de analisar a qualidade das práticas obstétricas de risco habitual, recomenda-se a utilização do instrumento validado neste estudo.

## CONCLUSÕES

Este trabalho permitiu a construção e disponibilização de um checklist e dois documentos de apoio com indicadores de validade de conteúdo para o monitoramento da assistência obstétrica realizada em maternidades.

A utilização dos três documentos poderá instrumentalizar instituições na avaliação da qualidade das ações direcionadas a mães e neonatos, considerando que a saúde materna e a saúde neonatal são indicadores importantes da qualidade de vida de determinada população. Nas últimas décadas, um movimento de importância de mulheres, órgãos governamentais e organizações internacionais vem lutando para assegurar a regionalização, a capacitação dos profissionais, a disponibilidade de recursos e qualificadas às mulheres e a seus filhos.

Recomenda-se a realização de estudos que utilizem o instrumento. Com isso, será possível avaliar seu desempenho na prática.

## Data Availability

Os dados de pesquisa só estão disponíveis mediante solicitação.
